# A comprehensive dataset for postpartum depression prediction among bangladeshi mothers: Sociodemographic, obstetric, and psychosocial factors with class imbalance insights

**DOI:** 10.1016/j.dib.2026.113210

**Published:** 2026-08-30

**Authors:** Shahriar Siddique Ayon, Shahidul Morsalin Jahin, Md. Ebrahim Hossain, Md. Saef Ullah Miah, Kazi Mostaq Hridoy, B.M. Mredul Arafin, Anik Dey Shaon, Md Rakibul Islam, M.F. Mridha

**Affiliations:** a Department of Computer Science, American International University Bangladesh, 1229, Dhaka, Bangladesh; b Department of Electrical & Computer Engineering, North South University, 1229, Dhaka, Bangladesh; c Department of Computer Science & Engineering, East West University, 1212, Dhaka, Bangladesh; d Department of Computer Science & Engineering, Southeast University, 1208, Dhaka, Bangladesh; e Department of Computer Science & Engineering, University of Chittagong, 4331, Chittagong, Bangladesh; f Department of Computer Science and Software Engineering, Malardalen University, Vastmanland, Sweden

**Keywords:** Postpartum depression, Maternal mental health, Machine learning, Cross-sectional study, Screening tools

## Abstract

Postpartum depression (PPD) is currently a serious mental disorder that occurs after childbirth. It affects the well-being of both the mother and the child. However, early screening is still limited in low- and middle-income countries like Bangladesh due to socio-economic challenges and social stigma. This paper presents a comprehensive and multidimensional dataset consisting of 766 participants to facilitate data-driven PPD research. The participating women were between the ages of 18–41 years. The data were collected through a cross-sectional study using face-to-face interviews and hospital records at LABAID Diagnostics, Pabna, Bangladesh, between March and June 2025. The dataset integrates 51 variables, including relevant factors such as sociodemographic factors, obstetric history, neonatal details, and psychosocial indicators. Maternal health was assessed using standardized screening tools: PHQ-2 and PHQ-9 for symptoms before and during pregnancy, and Edinburgh Postpartum Depression Scale (EPDS) for postpartum symptoms. Results indicated that the prevalence of depression during pregnancy among the participants was 40.47%. A key feature of this dataset is the clear reporting of class imbalance, which can be reused for statistical analysis, epidemiological studies, and validation of depression screening tools, making it a valuable resource for building robust machine learning (ML) and interpretable AI (XAI) models for early risk prediction. This study fills an important gap in publicly available, high-quality maternal health information in the South Asian context. It also provides the basis for evidence-based interventions and targeted support for at-risk mothers.

Specifications Table

Table 1. Dataset Specifications and Accessibility Profile.

**Table:** 

Subject	Health Sciences, Medical Sciences & Pharmacology
Specific subject area	Postpartum depression prediction among Bangladeshi women
Type of data	Raw, Processed
Data collection	This study employed a systematic, cross-sectional design at LABAID Diagnostics in Pabna, Bangladesh, between March and June 2025. All outpatients aged 18–41 were invited, and data were collected from 766 women through face-to-face interviews using a structured questionnaire on demographics, socioeconomic status, obstetric history, and mental well-being. Responses were securely recorded in a CSV file. Mental health was assessed with standardized tools, including PHQ-2, PHQ-9, and the Edinburgh Postnatal Depression Scale (EPDS). The dataset was rigorously reviewed for clinical validity before approval, and confidentiality was ensured by anonymizing all records with unique patient IDs.
Data source location	LABAID LTD. (Diagnostic), Pabna, Bangladesh (Latitude and Longitude: 24.0081,796°N,89.2389,675°E)
Data accessibility	Repository name: Mendeley Data Data identification number: 10.17632/nzgnsrgsg5.1 Direct URL to data: Bangladeshi Women Postpartum Depression Prediction Dataset - Mendeley Data Instructions for accessing these data: The dataset is licensed under CC BY 4.0 and is freely available for download without registration. Includes one UTF-8 encoded CSV file named Postpartum_depression_data.csv
Related research article	None

## 1 Value of the Data

• Enables investigation of how sociodemographic vulnerability interacts with obstetric and psychosocial factors, including pregnancy loss, spousal support, and household dynamics, to shape antenatal and postpartum depression risk in a South Asian low- and middle-income country (LMIC) context.
• Supports diverse secondary applications, including statistical modeling, epidemiological studies, validation of PHQ-2/PHQ-9/EPDS screening tools, and explainable or class-imbalance-aware machine learning for maternal mental health prediction.
• Explicit documentation of class imbalance and precise categorical encoding facilitates robust, generalizable predictive models for real-world postpartum depression scenarios.
• One of the largest open-access postpartum depression datasets from South Asia, providing culturally and contextually relevant data often missing from global repositories.
• Reusable for researchers, clinicians, and policymakers to conduct longitudinal, equity-focused studies and design evidence-based interventions to improve early screening and support in underserved populations.

## 2 Background

Postpartum depression (PPD) is a critical maternal mental health condition that typically emerges within the first year after childbirth and significantly affects the wellbeing of both mothers and infants. Globally, PPD prevalence ranges between 10 and 20%, but rates are considerably higher in low- and middle-income countries (LMICs) due to socioeconomic vulnerability, limited access to mental health services, and cultural stigma surrounding psychological disorders [1]. In South Asia, including Bangladesh, PPD has become an increasingly recognized public health concern. National estimates suggest that 18 to 35% of Bangladeshi mothers experience moderate to severe PPD symptoms, driven by factors such as financial stress, gender-based expectations, lack of spousal support, domestic violence, and inadequate postnatal care [2,3]. Despite its profound impact on maternal functioning, infant development, and long-term family wellbeing, early screening and diagnosis remain limited [4].

A major bottleneck in advancing data-driven maternal mental health research is the absence of structured, openly available, high-quality datasets that capture diverse predictors of PPD. Existing studies often rely on small sample sizes, clinically collected data, or narrowly focused surveys that emphasize either psychological or socioeconomic aspects, thereby failing to provide a comprehensive, multidimensional perspective [5].

Previous Research in South Asia, including Bangladesh, existing hospital based studies often rely on relatively small samples, typically 100 to 500 participants, report primarily aggregated findings, and provide limited access to underlying datasets, thereby restricting reproducibility and secondary analysis [6,7]. Furthermore, few studies integrate multidimensional factors, including household relationships, newborn characteristics, and longitudinal mental health attributes, within a single comprehensive dataset [6].

In Bangladesh, postpartum depression has been investigated using hospital-based and community-based cross-sectional studies. These studies have examined factors such as maternal age, socioeconomic status, pregnancy-related conditions, social support, domestic circumstances, and previous pregnancy experiences [8]. Another study has reported associations between postpartum depression and factors including inadequate social support, pregnancy-related difficulties, family circumstances, and maternal psychological experiences [9]. Research has also examined the performance of commonly used depression screening instruments, including the Patient Health Questionnaire-2 (PHQ-2), Patient Health Questionnaire-9 (PHQ-9), and Edinburgh Postnatal Depression Scale (EPDS), in maternal and postpartum populations [10,11].

The present dataset was developed to provide a multidimensional participant-level resource containing 51 variables collected from 766 Bangladeshi mothers. The dataset combines sociodemographic, socioeconomic, household, obstetric, neonatal, psychosocial, and depression-screening information. It also documents the distribution of depression-related screening outcomes, allowing researchers to investigate class imbalance in subsequent statistical and computational analyses.

PPD datasets are often imbalanced, with fewer positive cases due to underreporting, social stigma, and limited routine screening. Earlier datasets also frequently lack explicit documentation of class imbalance, limiting their utility for developing robust machine learning (ML) and explainable artificial intelligence (XAI) models for early risk prediction [6]. Such imbalance can bias learning algorithms toward the majority class, reducing their ability to identify relatively rare but clinically important PPD cases and compromising model generalizability. Addressing these limitations requires a well structured dataset that integrates sociodemographic, obstetric, behavioural, and psychosocial factors while transparently characterizing class distributions. Such a resource can facilitate advanced analytics, XAI methods, and imbalance aware ML techniques, supporting more reliable prediction and interpretable risk assessment.

The dataset is therefore intended primarily as a reusable data resource. It can support descriptive and inferential statistical analyses, epidemiological investigations, screening-tool evaluation, ML, XAI, and comparative studies of maternal mental health datasets. Ultimately, these capabilities can contribute to evidence based maternal mental health screening and targeted interventions that are better aligned with the Bangladeshi context.

## 3 Data Description

This dataset includes detailed information on demographics, socioeconomic and financial status, family and household relationships, pregnancy and childbirth history, medical and clinical conditions, newborn details, and mental health factors to better understand postpartum depression in women. The dataset includes women from urban, rural, and hospital outpatient areas, all within 24 months after giving birth.

Table 2 gives a clear overview of all variables, showing their names, descriptions, data types, and relevant units or categories. To facilitate navigation, the 51 variables are organized into seven thematic sections: (1) Demographic characteristics (e.g., age, residence, education level, marital status, family type, household size); (2) Socioeconomic and financial status (e.g., participant and husband occupation, monthly income before and after childbirth); (3) Parental and household relationships (e.g., relationships with in-laws, husband, and newborn, feeling about motherhood, received and needed support, abuse history, trust); (4) Obstetric and pregnancy history (e.g., pregnancy number, length, planning, prenatal checkups, fear of pregnancy, prior pregnancy loss); (5) Medical and clinical conditions (e.g., addiction, chronic diseases, mode of delivery, birth complications); (6) Psychological and mental health (e.g., PHQ-2 and PHQ-9 scores before and during pregnancy, EPDS score postpartum, anger, worry, ability to rest, feelings about regular activities); and (7) Newborn-related factors (e.g., newborn age, sex, illness, breastfeeding, maternal worry and sleep). Categorical and ordinal variables are retained as descriptive text labels to preserve readability and analytical flexibility while avoiding arbitrary numeric encoding. The dataset serves as the definitive codebook, providing all permissible response labels for each variable and allowing researchers to apply appropriate encodings based on their analytical methods.

**Table 2:** Definitions and categorical encoding of dataset variables.

Variable Name	Description	Data Type	Units / Categories
Patient ID	Unique identifier for each participant	Integer	1, 2, 3, ….
Age	Age of participant	Integer	18–41
Residence	Participant’s place of residence	String	City, Village
Education Level	Highest education attained	String	High School, University, College, Primary School, None
Marital status	Marital status of participant	String	Married, Divorced
Occupation before latest pregnancy	Participant’s occupation before latest pregnancy	String	Housewife, Student, Teacher, Other, Service, Business, Doctor
Monthly income before latest pregnancy	Monthly income before pregnancy	String	None, <5000, 3000–7000, 5000–10,000, 10,000–15,000, 10,000–20,000, 15,000–20,000, 15,000–25,000, 15,000–30,000, 20,000–30,000, >30,000, 3000–6000
Occupation After Your Latest Childbirth	Occupation after childbirth	String	Housewife, Student, Business, Other, Service, Teacher, Doctor
Current monthly income	Monthly income after childbirth	String	None, <5000, 3000–7000, 3000–6000, 5000–10,000, 10,000–15,000, 10,000–20,000, 15,000–20,000, 15,000–25,000, 15,000–30,000, 20,000–30,000, >30,000
Husband's education level	Highest education attained by husband	String	College, University, High School, Primary School, None
Husband's monthly income	Monthly income of husband	String	None, <5000, 5000–10,000, 10,000–20,000, 20,000–30,000, >30,000
Addiction	Addiction history of participant	String	None, Smoking, Drinking, Drugs
Total children	Number of children	String	One, Two, More than two
Disease before pregnancy	Chronic/non-chronic disease history	String	None, Non-Chronic Disease, Chronic Disease
History of pregnancy loss	Any previous pregnancy loss	String	None, Still-born Delivery, Miscarriage
Family type	Type of family	String	Joint, Nuclear
Number of household members	Total members in household	String	2 to 5, 6 to 8, 9 or more
Relationship with the in-laws	Quality of relationship with in-laws	String	Friendly, Good, Bad, Neutral
Relationship with husband	Quality of relationship with husband	String	Friendly, Good, Neutral, Bad
Relationship with the newborn	Quality of relationship with newborn	String	Very good, Good, Neutral, Bad
Relationship between father and newborn	Quality of father-newborn relationship	String	Very good, Good, Neutral, Bad
Feeling about motherhood	Emotional feeling about motherhood	String	Happy, Sad, Neutral
Recieved Support	Level of support received	String	High, Medium, Low
Need for Support	Participant’s perceived need for support	String	High, Medium, Low, None
Major changes or losses during pregnancy	Major life changes during pregnancy	String	Yes, No
Abuse	Experience of abuse	String	Yes, No, None
Trust and share feelings	Ability to trust and share feelings	String	Yes, No, None
Number of the latest pregnancy	Current pregnancy number	Integer	1, 2, 3, 4, 5, 7
Pregnancy length	Duration of current pregnancy	String	<5 months, 6 months, 7 months, 8 months, 9 months, 10 months, None
Pregnancy plan	Whether pregnancy was planned	String	Yes, No
Regular checkups	Attended prenatal checkups	String	Yes, No
Fear of pregnancy	Experienced fear during pregnancy	String	Yes, No
Diseases during pregnancy	Disease history during pregnancy	String	None, Non-Chronic Disease, Chronic disease
Age of newborn	Age of newborn	String	0 to 6 months, 6 months to 1 year, 1 year to 1.5 year, Older than 1.5 year
Age of immediate older children	Age of older siblings	String	None, 1 yr to 3 yr, 4 yr to 6 yr, 7 yr to 12 yr, 13 yr or more
Mode of delivery	Delivery method	String	Normal Delivery, Caesarean Section
Gender of newborn	Sex of newborn	String	Boy, Girl
Birth compliancy	Birth complications	String	Yes, No
Breastfeed	Whether newborn is breastfed	String	Yes, No
Newborn illness	Any illness in newborn	String	Yes, No
Worry about newborn	Whether participant worries about newborn	String	Yes, No
Relax/sleep when newborn is tended	Ability to rest while tending newborn	String	Yes, No
Relax/sleep when the newborn is asleep	Ability to rest while newborn sleeps	String	Yes, No
Angry after latest child birth	Experienced anger after childbirth	String	Yes, No
Feeling for regular activities	Feeling during daily activities	String	Tired, Worried, Afraid, None
Depression before pregnancy (PHQ2)	PHQ-2 score before pregnancy	String	Positive, Negative
Depression during pregnancy (PHQ2)	PHQ-2 score during pregnancy	String	Positive, Negative
PHQ9 Score	PHQ-9 total score	Integer	0–27
PHQ9 Result	PHQ-9 severity category	String	Minimal, Mild, Moderate, Moderately Severe, Severe
EPDS Score	Edinburgh Postnatal Depression Scale score	Integer	0–30
EPDS Result	EPDS severity category	String	Low, Medium, High

## 4 Experimental Design, Materials and Methods

### 4.1 Study setting and participants

Between March and June 2025, we conducted a cross-sectional study at LABAID Diagnostics, Pabna, a major healthcare center serving both urban and rural communities in Bangladesh [12]. During this period, all patients visiting the outpatient clinic were invited to participate. Eligible participants were aged 18–41, attending the hospital for routine care, medical checkups, or health assessments, and willing to provide informed consent. No financial or material compensation was offered; participants volunteered solely to contribute to future research on maternal mental health in Bangladesh. Patients with serious health conditions unrelated to depression were excluded from the study.

Our data collection was carried out by face-to-face interviews with the respondents directly entering them into a structured CSV file on a password keyed laptop. Hospital records were used to obtain clinical measurements. No personally identifiable information was noted in order to safeguard privacy. The study involved 766 women, 40.47% of whom were positive to test of depression during the pregnancy and 59.53% were negative who were used as a control group. The data is in CSV format with UTF-8 encoding, and has 766 rows and 51 columns, which is easy to understand and analyse as well as reproduce results. Following data collection, the dataset was carefully reviewed by doctors and hospital authorities who were keen on accuracy, consistency and clinical validity before being eventually approved.

### 4.2 Data collection procedures

The data was gathered among postpartum women with the help of a structured questionnaire which was created to reflect in detail such aspects of data as demographics, socioeconomic status, family background, pregnancy and obstetric history, medical and clinical conditions, newborn features, and psychological well-being.

The demographic and socioeconomic section included information such as age, residence, educational level, marital status, occupation, income, and household characteristics. The family and psychosocial sections covered family type, relationships with the husband and in-laws, relationship with the newborn, perceived support, need for support, emotional experiences related to motherhood, major changes or losses during pregnancy, experience of abuse, and the ability to trust and share feelings.

Depressive symptoms were assessed using three standardized self-report instruments, with the dataset retaining composite scores derived from the individual items. The Patient Health Questionnaire-2 (PHQ-2) is a two-item screening tool assessing depressive symptoms over the previous two weeks: (1) “Little interest or pleasure in doing things” and (2) “Feeling down, depressed, or hopeless.” Each item is rated on a 4-point scale from 0 (Not at all) to 3 (Nearly every day), yielding a total score of 0–6, with a score ≥3 indicating a positive depression screen. The PHQ-2 was administered for both pre-pregnancy and antenatal assessment, with results recorded as Positive or Negative.

The Patient Health Questionnaire-9 (PHQ-9) is a 9-item measure of depressive symptom severity over the previous two weeks, extending the PHQ-2 to include symptoms such as sleep disturbance, fatigue, appetite changes, concentration difficulties, psychomotor changes, and suicidal ideation. Each item is scored from 0 (Not at all) to 3 (Nearly every day), yielding a total score of 0–27. Severity is classified as Minimal (0–4), Mild (5–9), Moderate (10–14), Moderately Severe (15–19), or Severe (20–27). The PHQ-9 was administered before and during pregnancy.

The Edinburgh Postnatal Depression Scale (EPDS) is a 10-item self-report screening tool for postpartum depressive symptoms during the previous 7 days. It assesses mood, anxiety, sleep, coping, and self-harm thoughts, with items scored from 0 to 3 using item-specific response options, yielding a total score of 0–30. The dataset includes the composite EPDS score and a categorical outcome (Low, Medium, High), alongside clinical information such as delivery type, newborn age, birth complications, and maternal health factors.

Clinical information, including the type of delivery, newborn age, birth complications, and maternal health variables was noted in conjunction with self-reported information. The data were anonymized, and this made the dataset fit in research about the factors that contribute to postpartum depression. The Fig. 1 gives the overview of the dataset, which shows the various forms of information gathered by the participants and hospital records to do the analysis on postpartum depression.

**Fig. 1:**
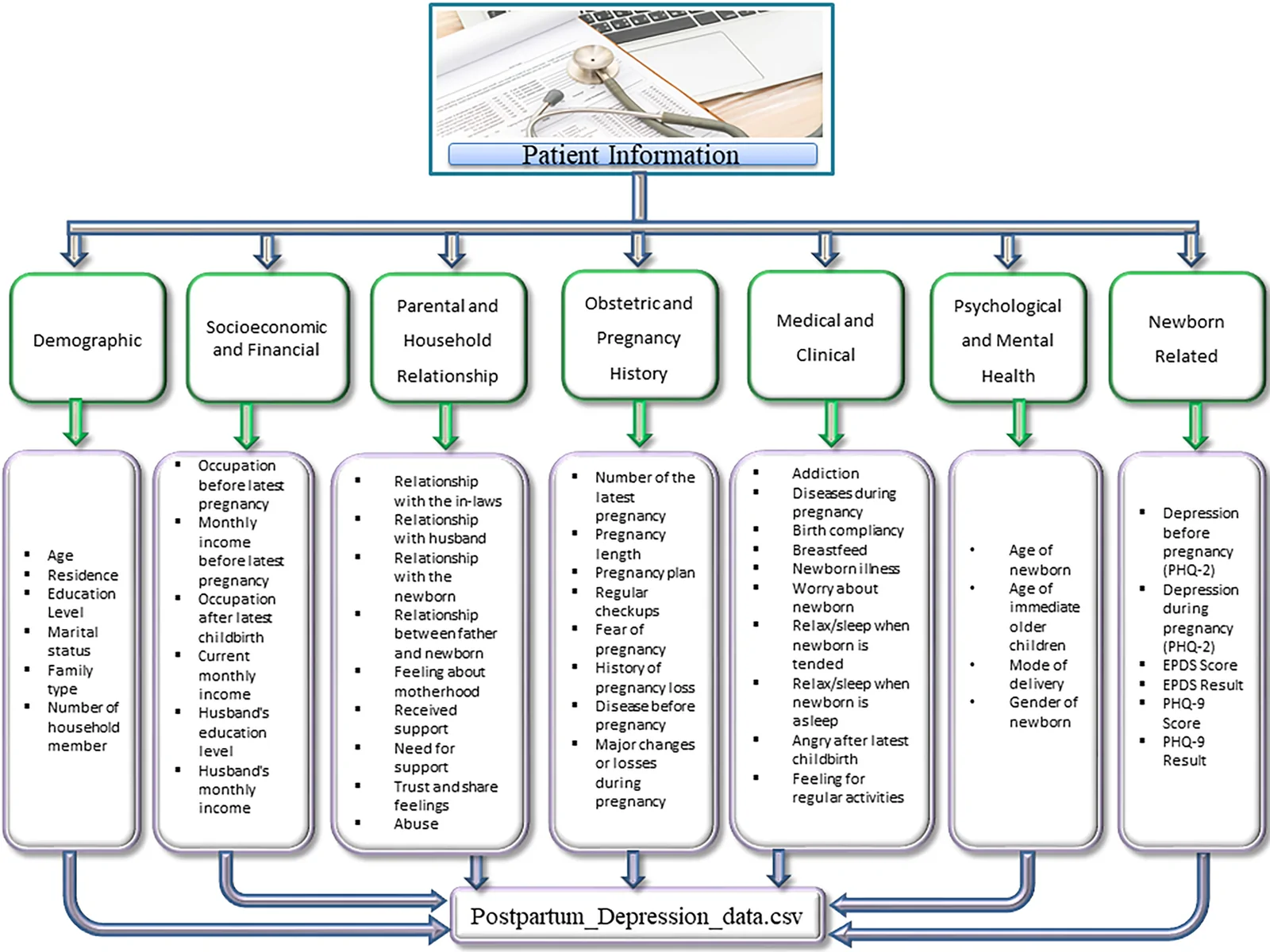
Conceptual framework of multidimensional predictors for postpartum depression.

Fig. 2 displays PHQ-9 scores plotted against maternal age (18–41 years), with individual participants represented as colored points corresponding to their PHQ-9 severity category: Minimal (blue), Mild (red), Moderate (green), Moderately Severe (orange), and Severe (purple). The figure provides a visual overview of the score distribution within each severity level across the observed age range.

**Fig. 2:**
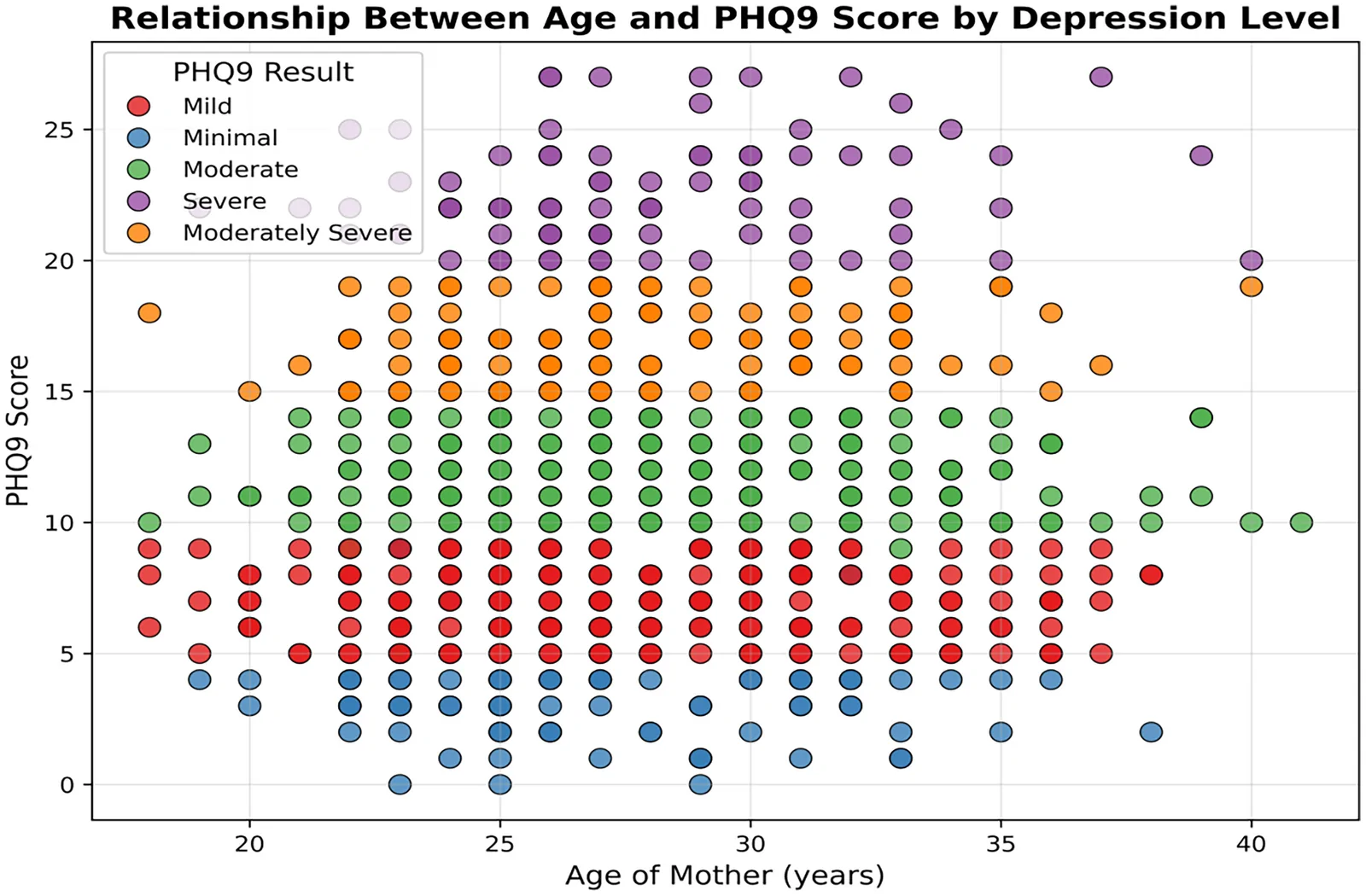
Distribution of PHQ-9 severity levels across the maternal age spectrum.

Fig. 3 presents EPDS scores (range 0–30) by pregnancy length category (10, 9, 8, 7, 6, <5 months, and none), with points colored according to EPDS severity level: Low (red), Medium (blue), and High (green). The figure illustrates the spread and clustering of postpartum depression scores within each gestational duration group.

**Fig. 3:**
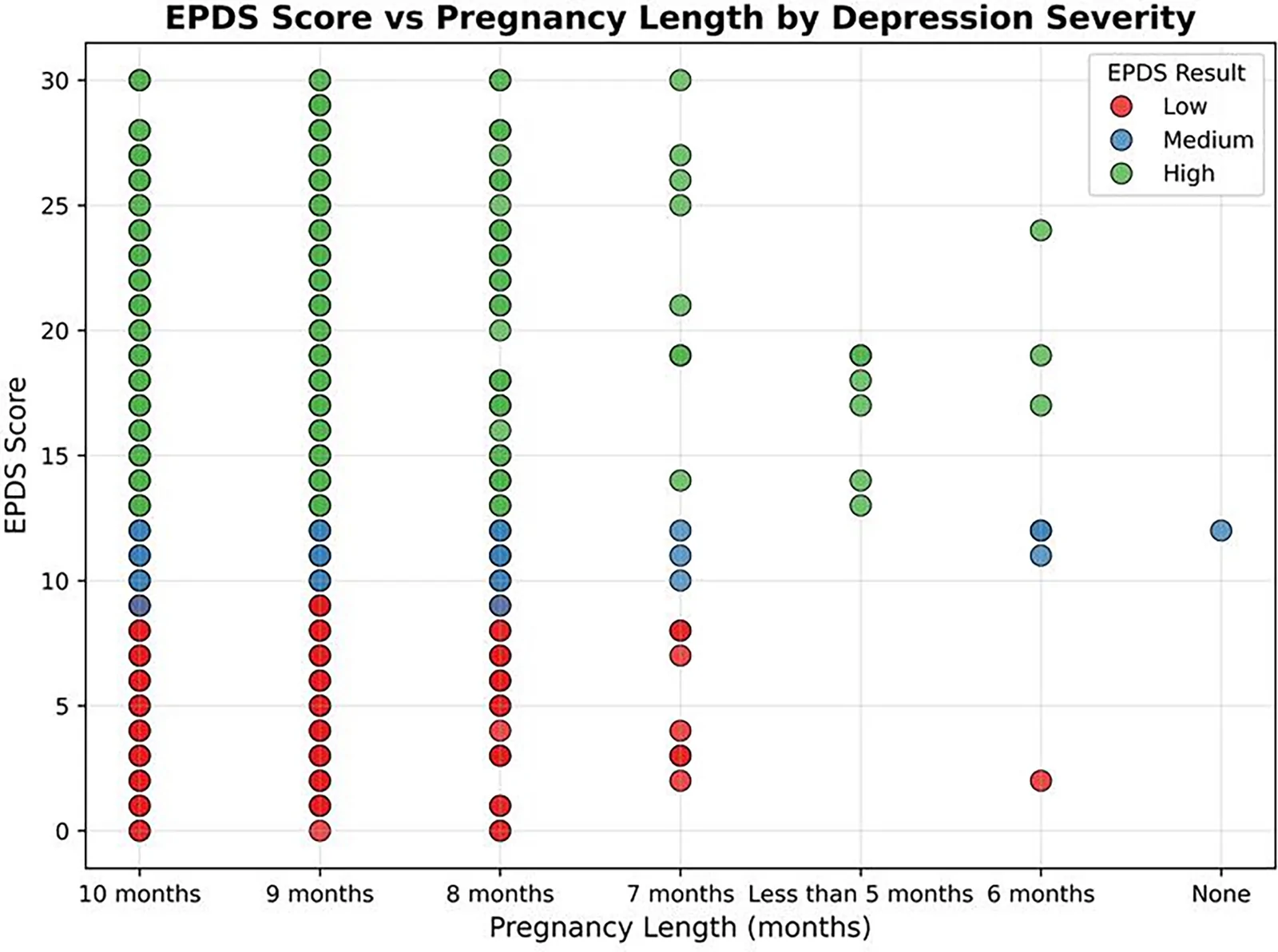
Correlation between gestational duration and postpartum depression severity (EPDS).

Fig. 4 shows the distribution of pregnancy loss history among women with negative and positive PHQ-2 antenatal depression screening results. The left bar represents women with negative screening, and the right bar represents women with positive screening. Segments are color-coded by loss type: miscarriage (blue), no prior loss (green), and still-born delivery (gray). Percentages within each bar indicate the proportion of participants in each screening group reporting the respective obstetric history.

**Fig. 4:**
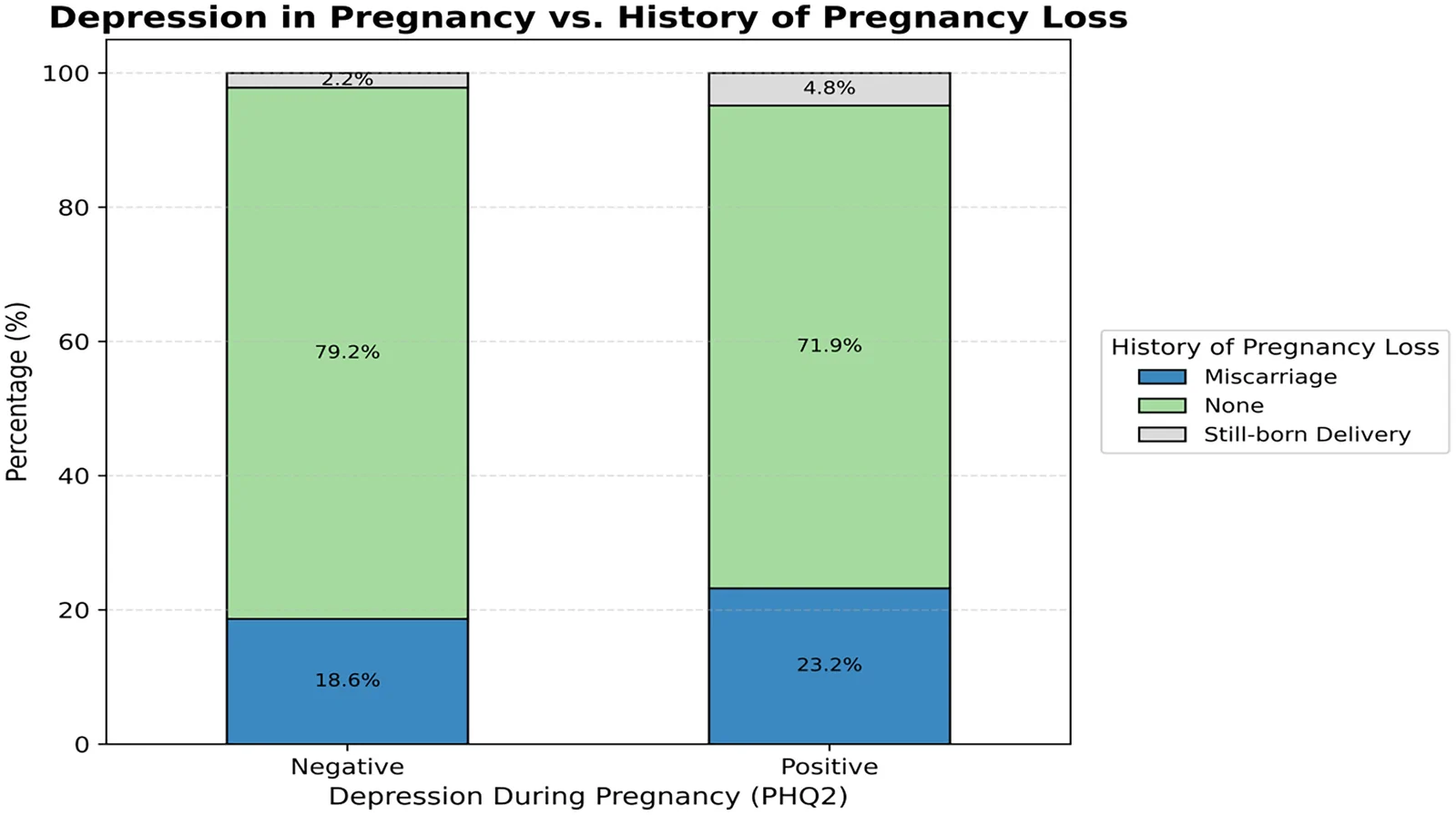
Prevalence of antenatal depressive symptoms (PHQ-2) relative to obstetric loss history.

### 4.3 Data preprocessing

The dataset was initially loaded and rigorously checked for completeness and consistency. There were no missing values, and items labeled ``None'' were determined to be genuine responses such as participant disagreement, non-applicability, or negative results, rather than missing data.

Potential outliers were cross-checked against authentic medical records to ensure accuracy. Then, comprehensive preprocessing was performed, which included the removal of duplicate records and the normalization of measurement units.

Categorical values were also harmonized to maintain consistent formatting, such as correcting capitalization ("Primary school" to ``Primary School''), refining spelling ("Housewife" to ``House wife''), adjusting medical terminology ("Chronic Disease" to ``Chronic disease''), and standardizing response labels related to pregnancy outcomes and interpersonal relationships ("Still-born delivery" to ``Still-born Delivery,'' ``Poor'' to ``Bad''). Minor variations, such as leading spaces (e.g., ``Severe''), were also addressed to ensure clean and dependable data.

All categorical and ordinal variables were recorded with descriptive labels to improve readability, with the option of subsequent recording in statistical or machine learning workflows. To ensure patient confidentiality, direct identifiers were eliminated, and each participant was given a unique Patient ID. The finalized dataset was exported as a CSV file encoded in UTF-8, and all items were individually cross-checked to assure data integrity.

## Limitations

This dataset has some important limitations that should be kept in mind when interpreting or reusing the data. The study is cross-sectional, meaning depressive symptoms were measured at a single point in time, which limits the ability to draw causal conclusions or examine how symptoms change before and after childbirth. In addition, participants were recruited from one healthcare center, so the findings may not fully represent mothers from other regions of Bangladesh or those with different levels of access to healthcare and social resources. Several variables, especially those related to mental health, family relationships, and personal experiences, rely on self-reported information and may be influenced by recall errors, social desirability, or reluctance to disclose emotional distress due to stigma. The lack of longitudinal follow-up also means that changes in depression severity over time and longer-term maternal or infant outcomes could not be assessed. Finally, cultural beliefs and social expectations within the Bangladeshi context may shape how emotional symptoms are understood and reported, which should be considered when using the dataset for cross-cultural or comparative studies.

## Ethics Statement

The study protocol was reviewed and approved by the medical and administrative authorities at LABAID LTD. (Diagnostic), Pabna, Bangladesh. All procedures involving human participants were conducted in accordance with the ethical standards of the institutional and/or national research committee, as well as the 1964 Helsinki Declaration and its later amendments. Participation was entirely voluntary, and no personally identifiable information was collected. To ensure the privacy and confidentiality of participants, all data were anonymized at the time of collection. Informed consent was obtained from every participant. This study specifically involved women aged 18 to 41. The resulting dataset was created solely for academic and research purposes and does not contain any sensitive or clinical identifiers that could be linked back to individual participants.

## CRediT authorship contribution statement

**Shahriar Siddique Ayon:** Conceptualization, Methodology, Visualization, Writing – original draft. **Shahidul Morsalin Jahin:** Methodology, Resources. **Md. Ebrahim Hossain:** Conceptualization, Resources, Writing – review & editing. **Md. Saef Ullah Miah:** Supervision, Writing – review & editing. **Kazi Mostaq Hridoy:** Visualization. **B.M. Mredul Arafin:** Writing – original draft. **Anik Dey Shaon:** Formal analysis. **Md Rakibul Islam:** Conceptualization, Methodology, Writing – original draft. **M.F. Mridha:** Supervision, Writing – review & editing.

## Data Availability

Mendeley DataBangladeshi Women Postpartum Depression Prediction Dataset (Original data)

## Appendix Supplementary materials

Image, application 1

Image, application 2

Image, application 3

Image, application 4
