# Supplementary material for: A comprehensive dataset for postpartum depression prediction among bangladeshi mothers: Sociodemographic, obstetric, and psychosocial factors with class imbalance insights
Source: Data Brief. 2026 Aug 30;69:113210. doi: 10.1016/j.dib.2026.113210 (PMC13733464; doi:10.1016/j.dib.2026.113210)
Supplement: Supplementary file 1 [file mmc1.pdf]

01766661901
LABAID LTD. (DIAGNOSTICS), PABNA
License No: HSM45122391 Reg. Code: HSM62542

LABAID Diagnostics
Home of Trust

Date: July 28, 2025

Certificate of Data Authenticity

This is to certify that the dataset on postpartum depression in Bangladeshi women, as collected by Shahriar Siddique Ayon, a student at American International University-Bangladesh (Student ID: 20-42265-1), has been reviewed and validated by LABAID (Diagnostics) Pabna. The data collection was conducted under our supervision over a four-month period, following ethical research guidelines and hospital data-handling protocols. We confirm that no personally identifiable information has been included in the dataset.

Dataset Summary

Total Participants: 766

Study Population: Postpartum women attending routine check-ups at LABAID (Diagnostics) Pabna
Data Collection Period: March 2025 – June 2025

Methodology: Structured, interviewer-administered survey
Key Domains Collected

Demographics & Socioeconomics: Age, residence, education, marital status, occupation & income (pre-/postpartum)

Obstetric & Clinical: Parity, gestational length, delivery mode, complications & loss history

Mental Health: Pre-pregnancy and Antenatal Depression Screening (PHQ-2), Postpartum Depression Evaluation (PHQ-9), Edinburgh Postnatal Depression Scale (EPDS)

Lifestyle & Support: Breastfeeding, newborn care, sleep patterns, stressors/abuse, social support

The primary aim of this dataset is to support machine learning, deep learning, and large language model analyses that identify risk factors for postpartum depression and inform simple, culturally appropriate interventions for Bangladeshi women.

We hereby affirm the authenticity of this data and grant permission for its use in academic research and peer-reviewed publications, on the condition that all ethical standards and patient confidentiality safeguards continue to be strictly observed.

[Signature] 28/07/25
Nitish Sen.
Branch Manager.
LABAID LTD. (Diagnostics), Pabna

Nitish Chandra Sen
Branch Manager
Labaid (Diagnostics)
Pabna

S.S Tower, Thana Para (In front of Joy Kalibari), Shalgaria, Pabna, 6600, Tel: 0731-63362, 0731-63382, Mobile : 01766661901, Email : info@labaidgroup.com, Web : www.labaidgroup.com
