# Supplementary material for: A comprehensive dataset for postpartum depression prediction among bangladeshi mothers: Sociodemographic, obstetric, and psychosocial factors with class imbalance insights
Source: Data Brief. 2026 Aug 30;69:113210. doi: 10.1016/j.dib.2026.113210 (PMC13733464; doi:10.1016/j.dib.2026.113210)
Supplement: Supplementary file 3 [file mmc3.pdf]

Study Title:
A Comprehensive Dataset for Postpartum Depression Prediction Among Bangladeshi Women: Sociodemographic, Obstetric, and Psychosocial Factors with Class Imbalance Insights

Principal Investigator:
Shahriar Siddique Ayon

Purpose of the Study

You are invited to participate in a research study aimed at understanding postpartum depression and related factors among mothers. The information collected will be used to create a dataset for scientific research and to develop machine learning models that may help improve maternal mental health assessment.

Participation

Participation in this study is **voluntary**. If you agree to participate, you will be asked to complete a questionnaire about:

- demographic information
- pregnancy and childbirth history
- social and family support
- emotional well-being and depression screening (PHQ-9 and EPDS scales)

The survey will take approximately **10–15 minutes** to complete.

Confidentiality

All information collected in this study will remain **strictly confidential**.

- No personal identifying information will be published.
- The dataset will be **anonymized** before being used for research purposes.
- The data may be used in scientific publications or shared in research repositories.

Risks and Benefits

There are **no significant risks** associated with participating in this study. The results of this research may help improve understanding and early detection of postpartum depression.

Voluntary Participation

Your participation is completely voluntary. You may withdraw from the study at any time without any negative consequences.

Consent Statement

By signing below, you confirm that:

- You have read and understood the information above.
- You voluntarily agree to participate in this study.

Participant Name: ______________________

Participant Signature: ______________________

Date: ______________________
