# Supplementary material for: A comprehensive dataset for postpartum depression prediction among bangladeshi mothers: Sociodemographic, obstetric, and psychosocial factors with class imbalance insights
Source: Data Brief. 2026 Aug 30;69:113210. doi: 10.1016/j.dib.2026.113210 (PMC13733464; doi:10.1016/j.dib.2026.113210)
Supplement: Supplementary file 4 [file mmc4.pdf]

Postpartum Depression Survey Questionnaire

Section A: Demographic Information

Q1. What is your age?
Options:

Q2. What is your place of residence?
Options: City / Rural

Q3. What is your highest level of education?
Options: High School / College / University / Postgraduate / Other

Q4. What is your marital status?
Options: Married / Single / Divorced / Widowed

Q5. What was your occupation before your latest pregnancy?
Options: Housewife / Student / Employed / Business / Other

Q6. What was your monthly income before your latest pregnancy?
Options: None / 5000–10000 / 10000–20000 / 20000–30000 / More than 30000

Q7. What is your occupation after your latest childbirth?
Options: Housewife / Student / Employed / Business / Other

Q8. What is your current monthly income?
Options: None / 5000–10000 / 10000–20000 / 20000–30000 / More than 30000

Q9. What is your husband's highest education level?
Options: High School / College / University / Postgraduate

Q10. What is your husband's monthly income?
Options: None / 10000–20000 / 20000–30000 / More than 30000

Section B: Family and Social Background

Q11. Do you have any addiction history?
Options: None / Smoking / Alcohol / Other

Q12. How many children do you have?
Options: 1 / 2 / 3 / More than 3

Q13. Did you have any disease before pregnancy?
Options: None / Chronic Disease / Non-Chronic Disease

Q14. Have you experienced pregnancy loss previously?
Options: Yes / No

Q15. What type of family do you live in?
Options: Joint / Nuclear

Q16. How many household members live in your home?
Options: 2–5 / 6–8 / 9 or more

Q17. How would you describe your relationship with your in-laws?
Options: Very good / Good / Neutral / Bad

Q18. How would you describe your relationship with your husband?
Options: Very good / Good / Neutral / Bad

Section C: Emotional and Social Support

Q19. How would you describe your relationship with your newborn?
Options: Very good / Good / Neutral / Poor

Q20. How would you describe the relationship between the father and the newborn?
Options: Very good / Good / Neutral / Poor

Q21. How do you feel about motherhood?
Options: Happy / Neutral / Worried / Afraid

Q22. What level of support have you received after childbirth?
Options: High / Medium / Low

Q23. How much support do you feel you need?
Options: High / Medium / Low / None

Q24. Did you experience any major changes or losses during pregnancy?
Options: Yes / No

Q25. Have you experienced any abuse?
Options: Yes / No

Q26. Do you feel comfortable trusting others and sharing your feelings?
Options: Yes / No

Section D: Pregnancy Information

Q27. What number pregnancy is this for you?
Options: 1 / 2 / 3 / More than 3

Q28. What was the length of your pregnancy?
Options: 9 months / 10 months / Other

Q29. Was this pregnancy planned?
Options: Yes / No

Q30. Did you attend regular prenatal checkups?
Options: Yes / No

Q31. Did you experience fear or anxiety during pregnancy?
Options: Yes / No

Q32. Did you experience any diseases during pregnancy?
Options: None / Chronic disease / Non-chronic disease

Section E: Newborn Information

Q33. What is the age of your newborn?
Options: 0–6 months / 6 months–1 year / More than 1 year

Q34. What is the age of your immediate older child (if applicable)?
Options: None / 0–6 months / 1–3 years / More than 3 years

Q35. What was the mode of delivery?
Options: Normal Delivery / Caesarean Section

Q36. What is the gender of the newborn?
Options: Boy / Girl

Q37. Were there any birth complications?
Options: Yes / No

Q38. Is the newborn breastfed?
Options: Yes / No

Q39. Does the newborn have any illness?
Options: Yes / No

Q40. Do you worry about your newborn's health?
Options: Yes / No

Section F: Postpartum Lifestyle and Feelings

Q41. Are you able to relax or sleep when someone else takes care of the newborn?
Options: Yes / No

Q42. Are you able to relax or sleep when the newborn is asleep?
Options: Yes / No

Q43. Do you feel angry after your latest childbirth?
Options: Yes / No

Q44. How do you feel about performing regular daily activities?
Options: Tired / Worried / Afraid / Normal

Section G: Depression Screening

Q45. Did you experience depression before pregnancy (PHQ-2 screening)?
Options: Positive / Negative

Q46. Did you experience depression during pregnancy (PHQ-2 screening)?
Options: Positive / Negative

Q47. PHQ-9 Score (Postpartum Depression Assessment)
Scale: 0–27

Q48. PHQ-9 Result (Depression Severity)
Options: Minimal / Mild / Moderate / Moderately Severe / Severe

Q49. EPDS Score (Edinburgh Postnatal Depression Scale)
Scale: 0–30

Q50. EPDS Result (Depression Severity Category)
Options: Low / Medium / High
